# SARS-CoV-2 delta and omicron variants alter trophoblast cell fusion and syncytiotrophoblast dynamics: new insights into placental vulnerability

**DOI:** 10.1038/s41419-025-08016-x

**Published:** 2025-10-07

**Authors:** Manel Essaidi-Laziosi, Catia Alvarez, Michal Yaron, Pascale Sattonnet-Roche, Kenneth Adea, Mélanie Cornut, Christine Wuillemin, Meriem Bekliz, Yoann Sarmiento, Chloe Gibson, Laurent Kaiser, Anne-Laure Rougemont, Isabella Eckerle, Marie Cohen

**Affiliations:** 1 Geneva Centre for Emerging Viral Diseases, Geneva University Hospitals and Faculty of Medicine, University of Geneva, Geneva, Switzerland; 2 Department of Medicine, Faculty of Medicine, University of Geneva, Geneva, Switzerland; 3 Service of Gynecology, Department of Woman, Child and Adolescent, Geneva University Hospitals, Geneva, Switzerland; 4 Department of Pediatrics, Gynecology and Obstetrics, Faculty of Medicine, University of Geneva, Geneva, Switzerland; 5 Pediatric and Fetoplacental Pathology Unit, Division of Clinical Pathology, Geneva University Hospitals, Geneva, Switzerland

**Keywords:** Infection, Experimental models of disease

## Abstract

Pregnancy is associated with an increased risk of severe COVID-19. In addition, SARS-CoV-2 infection during gestation has been linked to adverse obstetrical outcomes and placental abnormalities. Nevertheless, the susceptibility of early trophoblast cells to SARS-CoV-2 and the potential consequences of infection on trophoblast function remain unclear. In this study, we assessed the permissiveness of first trimester trophoblast cells to SARS-CoV-2 infection and its impact on trophoblast cells fusion. To address this, we isolated primary cytotrophoblast (CTB) cells from first trimester human placentas and allow their differentiation into STB in vitro. These cells were infected with SARS-CoV-2 variants of concern, including Delta and Omicron (BA.1, BA.2, BA.5). Viral replication was assessed by RT-qPCR and immunofluorescence, while host cell responses, including expression of viral entry receptors and innate immunity genes, were measured by RT-qPCR. Trophoblast fusion was evaluated by staining and calculating the fusion index. In parallel, placental tissues from SARS-CoV-2-infected pregnancies were analyzed by immunohistochemistry to quantify syncytial knots (SK) formation in vivo. Our results demonstrate that both first trimester CTB and STB are permissive to SARS-CoV-2 infection in a variant- and donor-dependent manners, with Delta exhibiting higher replication efficiency compared to Omicron variants. In STB, viral replication did not correlate with the induction of entry receptors or type III interferon responses. However, in CTB, viral replication was significantly associated with enhanced cell fusion. In parallel, an increased number of SK was observed in infected placental areas in vivo compared to non-infected regions from the same placenta and to gestational age-matched controls. Altogether, these in vitro and in vivo results suggest that SARS-CoV-2 infection in early pregnancy may alter STB turnover, potentially contributing to placental dysfunction and adverse pregnancy outcomes.

## Introduction

Association between COVID-19 infections and wide range of clinical adverse outcomes during gestation and post-partum period has been reported, as reviewed in [1, 2], affecting (i) the fetus, such as miscarriage, (ii) neonate like stillbirth, and (iii) the mother, with higher incidence of intensive care admission and death compared to age-matched non-pregnant women [3], as well as higher risk of premature delivery and pre-eclampsia. SARS-CoV-2 placentitis may affect both symptomatic and asymptomatic infected pregnant women, and combines chronic histiocytic intervillositis, massive perivillous fibrin deposition, and trophoblast necrosis [1, 4].

SARS-CoV-2 considerably evolved genetically since its emergence, giving rise to a range of variants with different disease severity and transmission capacities [5]. Across successive waves of SARS-CoV-2 variants of concern, several studies have reported a progressive increase in hospitalization rates among pregnant women, with higher risks observed during the Alpha variant wave compared to the ancestral strain, and a further rise of this rate during the subsequent Delta variant wave in 2021 [6–8]. In contrast, the risk of preterm birth and admission to maternal critical care has decreased during the Omicron- versus the Delta- dominant period [8]. Intrinsic phenotypic differences were observed between viruses, such as Delta and Omicron BA.2 and BA.5, but not BA.1, showed an enhanced fusogenicity compared to the ancestral virus. At the same time, with the emergence of Omicron, underlying population immunity has increased after the emergence of Omicron. Both changes in viral properties and immunity impact virus transmissibility and pathogenicity.

The exact mechanisms responsible for pregnancy complications due to SARS-CoV-2 remain unclear. However, enhanced inflammatory responses are thought to be a major driver of the placenta pathologies observed during SARS-CoV-2 maternal infections. Massive infiltration of immune cells and high induction of pro-inflammatory cytokines could be found at the maternal-fetal interface in SARS-CoV-2 infected pregnant women, even in the absence of placenta or fetus infections [9]. Although less common (maximum 8.5% according to a recent systematic review [10]), vertical transmission has been reported. Viral material could be detected in placental tissue and was localized mainly in syncytiotrophoblast (STB), but also in Hofbauer and stroma cells [4, 11]. Placental infections have been associated with higher risk of adverse obstetrical outcomes [12]. Although not surprising considering the critical role of this maternal-fetal interface organ in protecting, oxygenizing, and nourishing the developing fetus, a better understanding of the cause of these adverse clinical outcomes is still needed.

While the presence of SARS-CoV-2 was demonstrated in placenta of infected women, it is still unclear if it could efficiently replicate in this tissue. During the early steps of SARS-CoV-2 infection, the interplay between virus and placenta response might be determinant for viral replication and overall outcome of pregnancy. This includes innate immune response mediated by the activation of interferon (IFN) pathways and host factors involved in virus entry, such as Angiotensin-converting enzyme 2 (ACE-2) and transmembrane serine protease 2 (TMPRSS2) [13]. Tallarek et al. failed to demonstrate replication of SARS-CoV-2 in placental explants [14]. However, Fahmi et al. reported that SARS-CoV-2 could infect and propagate in term placental section explants in association with the expression of ACE-2, but not with TMPRSS2 [15]. As maternal-fetal virus transmission and pathogenesis might be dependent on gestational age, similar studies from earlier stage of gestation are relevant but limited by the restricted accessibility of placenta before term. Alternatively, trophoblast stem cells were recently used to generate an in vitro SARS-CoV-2 infection model of placenta [16].

Placental formation and development are complex multistep processes which start with blastocyst implantation in the endometrium. At the feto-maternal interface, trophoblast cells differentiate along either the villous or the extravillous pathway [17]. In the latter, extravillous cytotrophoblasts (CTBs) proliferate and differentiate into an invasive phenotype. These cells invade decidual stromal compartments as well as spiral arteries of the decidua and the proximal third of the myometrium [18]. In the villous pathway, villous CTB (vCTB) remains in the fetal compartment, fuses, and differentiates to form the syncytiotrophoblast (STB) [19]. Then, vCTB fuses with overlying STB to bring fresh cellular components to the STB. To maintain homeostasis, apoptotic material of STB is packed into syncytial knots (SK) and then released into maternal circulation. Controlled CTB-STB fusion and STB turnover are thus crucial to maintain the integrity of the placental barrier. An increased number of SK has been suggested to induce an inflammatory response by the mother and may lead to adverse obstetrical outcomes such as preeclampsia, a hypertensive disorder of pregnancy, and a major cause of maternal mortality and morbidity [20–23]. Examination of placenta obtained from patients with and without SARS-CoV-2 infection in pregnancy showed an association between SARS-CoV-2 infection and features of maternal vascular malperfusion and accelerated villous maturation [24, 25]. The strongest associations were found with infection in the eras of the Delta and Omicron variants [25] and early in gestation [24]. In line with an accelerated villous maturation, several studies also reported an increase of SK formation in placenta in late stage of pregnancy analysed from SARS-CoV-2 infected pregnant women [26–28]. These findings suggest that SARS-CoV-2 infection could alter STB turnover and are supported by a recent in vitro study demonstrating that SARS-CoV-2 can induce trophoblast fusion and apoptosis [29]. However, studies using human trophoblast stem cells (hTSC) to determine the effect of SARS-CoV-2 infection on STB differentiation observed a decrease in cell fusion with SARS-CoV-2 infection [30]. These discrepancies could be due to the different trophoblast models used for infection and cell fusion studies. Indeed, immortalized trophoblast cell lines, especially the BeWo cell line, are often considered as a model for syncytialisation [31]. However, these models require biochemical reagents to induce cell fusion, which may differ from the spontaneous fusion of trophoblast observed in vivo or in vitro. hTSCs are another interesting model where the cells differentiate into different trophoblast cells, including STB [31]. Nevertheless, they have not undergone rigorous comparison with in vivo or in vitro trophoblast cells.

CTBs isolated from trophoblast, where cells at different gestational stage spontaneously fuse and differentiate into STB, with a fusion index of 90% within 72 h are considered to be a relevant model for studying trophoblast fusion [31]. This model is also considered as a valuable model in placental infection studies. However, the usage of first trimester CTB is limited by the difficulty obtaining patient samples.

In this work, we evaluate replicative capacity of SARS-CoV-2 Delta variant and Omicron BA.1, BA.2, and BA.5 in primary first trimester CTB and STB. We also investigated host response induction in the context of infection and the effect of SARS-CoV-2 infection on trophoblast cell fusion.

## Methods and materials

### SARS-CoV-2 variants

Viral stocks of SARS-CoV-2 Delta and Omicron BA.1, BA.2, and BA.5 have been produced after isolation from left over of anonymized clinical samples collected at University Hospitals of Geneva as described previously [32]. All virus isolations, viral stocks production and titrations have been performed in Vero-E6 derived cells overexpressing TMPRSS2 as already described [32, 33]. All information about variants is summarized in table S1.

### Trophoblast cells isolation and culture

This study has been approved by the Cantonal Commission for research ethics -ID 2022-00873. Informed written consent to participate in the study was obtained from patients who voluntarily decided to interrupt their pregnancy (9-11 weeks of gestation) at the outpatient gynecology consultations of Geneva University Hospitals. First trimester CTBs were isolated as previously described [34]. Briefly, trophoblast tissues were isolated and enzymatically digested with a Difco Trypsin solution (BD, Le pont de Claix, France). After separation in a Percoll gradient (GE Healthcare, Uppsala, Sweden), CTBs were immunopurified using monoclonal mouse anti-human CD45 immobilized antibodies (Dako, Glostrup, Denmark). After immune-purification cells were resuspended in Dulbecco’s modified Eagle’s medium (DMEM; Gibco, Invitrogen, Basel, Switzerland) supplemented with 10% FBS and 0.05 mg/ml gentamycin. Cells were then seeded either in 96-well (1.10^5^ cells/well) or 24-well (1.5.10^6^ cells/well) plates for, respectively, CTB or STB infection assays. They were incubated 24 h (for CTB infections) or 72 h to allow cell fusion (for STB infection) at 37 °C and 5% CO_2_ before virus infection. All details about placenta cells from each donor and information about the corresponding analyses are summarized in table S2.

### Virus infection of trophoblastic cells

Infections of trophoblast with SARS-CoV-2 were performed at 37 °C and 5% CO_2_ and an approximative multiplicity of infection (MOI) of 0.1-0.2. These MOIs (see Table S1) were defined according to the optimal kinetics of each virus shown in human airway epithelia [35–38] and preliminary tested in placenta cells (data not shown). STB and CTB were inoculated with 100 μL of virus diluted in 100 μL of culture medium, DMEM supplemented with 2% fetal bovine serum (FBS, Gibco), 2mM L-glutamine (Gibco), 1% penicillin-streptomycin (Gibco) for 1 h. They were then washed and overlaid with 500 μL (STB) or 200 μL (CTB) with the same culture medium and incubated at 37 °C, 5% CO_2_. For STB infections, supernatant was collected at 24 or 96 h post infection (hpi), and cells were lysed using the lysis buffer of NucliSens easyMAG (BioMérieux) at 96hpi. Two days after CTB infections, supernatant was collected, and cells were fixed using 6% paraformaldehyde (PAF) at least 1 h at room temperature (RT).

### Virus replication

Virus replication was assessed by viral RNA quantification and virus titration. Viral RNA was extracted from the infection supernatant collected at different time points and used for quantitative real time PCR (RT-qPCR) using SuperScript™ III Platinum™ One-Step qRT-PCR Kit (Invitrogen) in a CFX96 Thermo Cycler (BIORAD) and E-gene targeting primers and probe [30]. Data was collected and analyzed using Bio-Rad CFX maestro software (BIORAD).

Infectious titer of SARS-CoV-2 was also assessed by focus-forming assay as previously described [35]. Briefly, after 1-h inoculation using serially diluted supernatant collected from STB infections at 4dpi, Vero E6 cells (40,000) were overlayed by prewarmed DMEM (10%FBS, 2mM L-glutamine, 1%penicillin-streptomycin all from Gibco) mixed (1:1) with 2.4% Avicel and incubated 24-h at 37 °C and 5% CO_2_. Cells were then fixed (6% PAF at least 1-h, RT), permeabilized (0.1%Triton X-100 and blocked with 1% bovine serum albumin (Sigma)), and incubated 1-h at RT with a monoclonal anti-SARS-CoV N antibody (JS02 produced by Geneva Antibody facility at the Faculty of Medicine of Geneva). The cells were finally incubated 30 min at RT with the peroxidase-conjugated (HRP) secondary antibody (Jackson ImmunoResearch, 109-036-09), and True Blue HRP substrate (Avantor) was used to visualize foci. Images were acquired using ELISPOT reader to count foci and determine the number of focus-forming units per mL (FFU/mL) for each sample.

### Host response

Induction of interferons (IFN-α, IFN-β, and IFN-λ), ISG15 (Interferon stimulated gene 15), pro-inflammatory cytokines, namely interleukin-6 (IL-6) and Tumor Necrosis Factor-alpha (TNF-α), and virus entry factors ACE-2 and TMPRSS2 were assessed from SARS-CoV-2 infected STB lysates collected at 96hpi. After RNA extraction, mRNA was semi-quantified by real-time RT-PCR using commercially available gene expression assay kits (Life Technology). Gene induction in infected tissues was represented in log10 fold change (FC) relative to mock-infected cells from the same donor and normalized to a housekeeping gene, RNAseP (Life Technology). The fold change (FC) was calculated as follows: FC = 2exp(normalized CT_mock – normalized CT_virus), where CT: cycle threshold obtained from Taqman.

### Immunofluorescence

Mock- and SARS-CoV-2 infected STB were PFA-fixed at 96hpi, washed with PBS, permeabilized, and co-immunostained with antibodies targeting Cytokeratin 7 (CK7; clone OV-TL, DAKO), as a trophoblast cell marker, and Nucleoprotein (N) SARS-CoV-2 (Rockland 200-401-50) to detect infected cells. Nuclei were stained with DAPI (4’, 6-diamino-2-phenylindole). Alexafluor anti-rabbit 488 antibody (A11032, Life 185 Technologies), and alexafluor anti-mouse 647 186 (A31571, Invitrogen) were used as secondary antibodies. Images were acquired using Zeiss LSM700/LSM800 Meta confocal microscope with a 63.6/1.4 objective.

### Fusion index

Fusion index (FI) was determined by immunocytochemistry from infected CTB at 48hpi. After Mayer’s Hemalun staining, images were required using EVOS microscope and syncytia were counted from triplicates of each infection condition. Non-infected CTB were used as controls. The percentage of FI was calculated as follows: FI = 100 X [(number of nuclei in syncytia- number of syncytia)/ total number of nuclei] and represented in percentage relative to FI in non-infected cells from the same donor.

### Staining of SARS-CoV-2 positive placentae and determination of SK

**Fig. 1:**
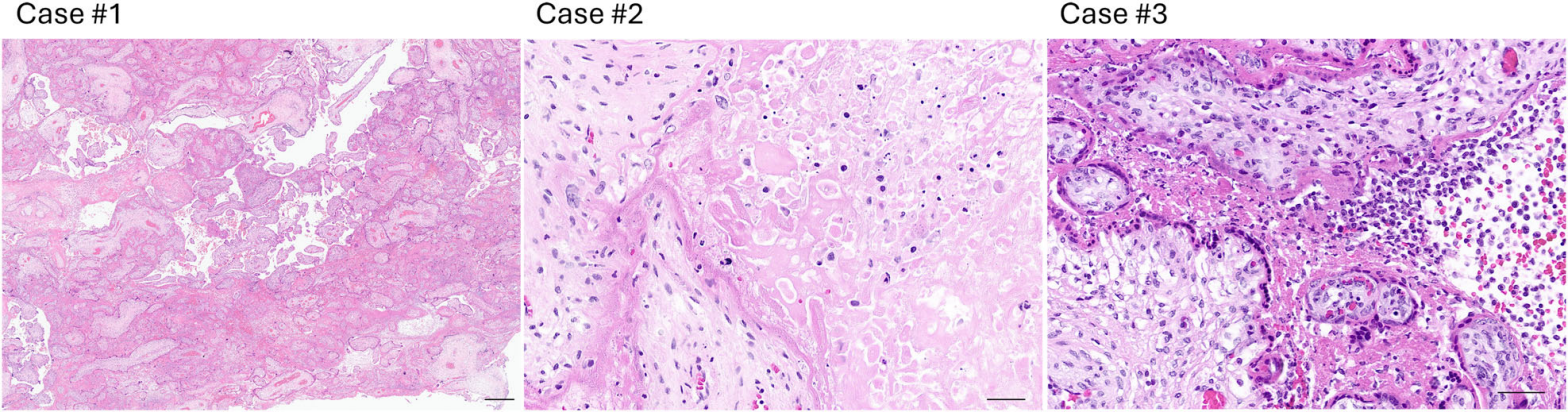
Main histological findings (H&E). The three early second trimester placentas with SARS-CoV-2 placentitis showed massive perivillous fibrin deposition, together with trophoblast necrosis in Case #2 and chronic histiocytic intervillositis in Case #3 (H&E; Case #1 - scale bar = 1 mm, Case #2 scale bar = 100 mm, Case #3 scale bar = 100 mm).

Placentas from three non-vaccinated COVID-19 positive patients with immunohistochemical confirmation of placental SARS-CoV-2 infection were assessed with the agreement of our local ethical committee. In absence of available material from the first trimester, placentas from the early second trimester of pregnancy (18 gestational weeks (GW)+ 1 day (d), 21 GW+ 1 d, and 24 GW+ 6 d respectively) were included. All three pregnancies resulted with intrauterine fetal demise. Expulsion took place between November 2020 and December 2021, corresponding to waves where Alpha (end of 2020-mid2021), then Delta (mid-2021-early 2022) variants were dominant. Placental histology was consistent with SARS-CoV-2 placentitis, showing massive perivillous fibrin deposition, with fibrin encasing more than 80% of the villi (Fig. 1).

The clinical characteristics and main placental findings are summarized in Table 1.

**Table 1:** Clinical characteristics and placenta findings.

Case#	GA (GW)	MA (y)	Covid-19 vaccination	SARS-CoV-2 infection	Method of detection	Maternal symptoms	Pregnancy outcome	Fetal sex	PW (g)	Main placenta pathology
1	18 GW + 1 d	28	No	d9 Covid+	PCR	Mild	IUFD	F	70 (P50 = 92 ± 17)	MPFD, trophoblast necrosis
2	24 GW + 6 d	40	No	d13 Covid+	Nasal swab	Asymptomatic	IUFD	M	183 (P50 = 189)	MPFD, intraparenchymal hematoma
3	21 GW + 1 d	43	No	On admission (d9 symptoms)	PCR, N501Y mutation	Mild	IUFD	F	110 (P50 = 143, P10 = 114)	MPFD, CHI

*GA* Gestational age, *GW* Gestational weeks, *MA* maternal age, *y* years, *d* day, *PCR* polymerase chain reaction, *IUFD* intrauterine fetal demise, *F* female, *M* male, *PW* placental weight, *g* grams, *P* percentile, *MFPD* massive perivillous fibrin deposition, *CHI* chronic histiocytic intervillositis.

For SK counts, 5 serial 3-µm thick sections were prepared from one selected formalin-fixed paraffin-embedded block from each of the three second trimester placentas. Immunohistochemistry was performed on the first and on the last sections, using a SARS-CoV-2 nucleocapsid antibody (Bio SB, clone BSB-134). Serial sections 2, 3, and 4 were stained with hematoxylin-eosin (H&E) (Fig. 1S).

SK counts were performed on the serial H&E placental sections. SK were defined as sessile aggregations of nuclei appearing and disappearing through the serial sections and seen in two or more sections. The analysis of SK release was then categorized on individual villi depending on the SARS-CoV-2 staining. Negative: villi showing no reactivity to SARS-CoV-2 nucleocapsid; mild: focal or weak SARS-CoV-2 staining; Positive: circumferential strong SARS-CoV-2 positivity. In the three placentas, SK counts were performed on five distinct villi from regions representing each of the three SARS-CoV-2 staining patterns (negative, focal/mild, and positive). The mean number of SK per square centimeter of villous surface area was calculated and reported. SK results are reported in Table 2 and compared to reference numbers [39] when feasible (reference data unavailable at 18 weeks of gestation).

**Table 2:** Determination of SK release of three early second trimester placenta from COVID19 positive pregnant patients.

Case #1 18 GW	SARS-CoV-2 NC	Circ (mm)	Surface (mm^2^)	Histology	Perivillous fibrin	SCT necrosis	Caryorrhexis	Villous fibrosis	Vessels	SK	% of villi with SK	Reference
V1	Negative	2.75	0.157	↑Hofbauer cells	No	No	No	No	Preserved	No	34%	NA
V2	Negative	0.71	0.028	—	No	No	No	No	Preserved	No		
V3	Negative	1.84	0.101	—	No	No	No	No	Preserved	No		
V4	Negative	0.897	0.04	Edema	No	No	No	No	Preserved	1		
V5	Negative	0.873	0.051	↑Hofbauer cells	No	No	Yes	No	Collapsed	No		
V6	Focal/mild	1.97	0.106	Edema, linear calcifications	0.087	No	Yes	Mild	Partially collapsed	5		
V7	Focal/mild	1.71	0.158	Edema	80% encased	Yes	No	No	Preserved	4		
V8	Focal/mild	1.28	0.119	Linear calcifications	50% encased	No	Yes	Mild	Preserved	2		
V9	Focal/mild	1.95	0.1	—	0.15	No	No	Mild	Preserved	5		
V10	Focal/mild	5.39	0.767	Edema, linear calcifications	No	No	No	No	Preserved	No		
V11	Positive	1.99	0.115	“Infarct”	Encased	Yes	No	Yes	Open lumen	6		
V12	Positive	1.18	0.055	“Infarct”	Encased	Yes	No	Yes	Open lumen	3		
V13	Positive	2.88	0.211	“Infarct”	Encased	Yes	No	Yes	Collapsed	2		
V14	Positive	3.1	0.234	“Infarct”	Encased	Yes	No	Yes	Partially collapsed	6		
V15	Positive	2.79	0.348	“Infarct”	Encased	Yes	No	Yes	Highly collapsed	7		

Case #2 24 GW	SARS-CoV-2 NC	Circ (mm)	Surface (mm^2^)	Histology	Perivillous fibrin	SCT necrosis	Caryorrhexis	Fibrosis	Vessels	SK	% of villi with SK	Reference
V1	Negative	1.31	0.124	—	No	Yes	No	Mild	Preserved	3	43%	8.6% (7–11%)
V2	Negative	0.966	0.029	Linear calcifications	No	No	No	No	Preserved	0		
V3	Negative	1.14	0.041	Linear calcifications	No	No	No	No	Preserved	1		
V4	Negative	1.65	0.089	—	No	No	No	No	Preserved	1		
V5	Negative	2.24	0.149	—	No	No	No	No	Preserved	0		
V6	Focal/mild	2.41	0.118	—	No	No	Yes	Mild	Partially collapsed	1		
V7	Focal/mild	2.52	0.176	CHI	No	No	No	No	Partially collapsed	4		
V8	Focal/mild	3.36	0.28	CHI	50% encased	Mild	No	No	Preserved	8		
V9	Focal/mild	0.731	0.035	CHI	20% encased	No	No	No	Preserved	5		
V10	Focal/mild	1.36	0.087	CHI	No	No	No	No	Preserved	2		
V11	Positive	2.49	0.168	“Infarct”	Encased	Yes	No	Yes	Open lumen	4		
V12	Positive	1.17	0.052	“Infarct”	Encased	Yes	No	Yes	Open lumen	4		
V13	Positive	0.917	0.028	—	50% encased	Mild	No	Yes	Collapsed	5		
V14	Positive	1.42	0.09	—	Encased	Yes	No	Yes	Open lumen	4		
V15	Positive	0.843	0.042	—	50% encased	Mild	No	No	Open lumen	3		

Case #3 21 GW	SARS-CoV-2 NC	Circ (mm)	Surface (mm^2^)	Histology	Fibrin	SCT necrosis	Caryorrhexis	Fibrosis	Vessels	SK	% of villi with SK	Reference
V1	Negative	1.31	0.124	—	No	Yes	No	Mild	Preserved	3	35%	5.5% (5–7%)
V2	Negative	0.966	0.029	Linear calcifications	No	No	No	No	Preserved	0		
V3	Negative	1.14	0.041	Linear calcifications	No	No	No	No	Preserved	1		
V4	Negative	1.65	0.089	—	No	No	No	No	Preserved	1		
V5	Negative	2.24	0.149	—	No	No	No	No	Preserved	0		
V6	Focal/mild	2.41	0.118	—	No	No	Yes	Mild	Partially collapsed	1		
V7	Focal/mild	2.52	0.176	CHI	No	No	No	No	Partially collapsed	4		
V8	Focal/mild	3.36	0.28	CHI	50% encased	Mild	No	No	Preserved	8		
V9	Focal/mild	0.731	0.035	CHI	20% encased	No	No	No	Preserved	5		
V10	Focal/mild	1.36	0.087	CHI	No	No	No	No	Preserved	2		
V11	Positive	2.49	0.168	“Infarct”	Encased	Yes	No	Yes	Open lumen	4		
V12	Positive	1.17	0.052	“Infarct”	Encased	Yes	No	Yes	Open lumen	4		
V13	Positive	0.917	0.028	—	50% encased	Mild	No	Yes	Collapsed	5		
V14	Positive	1.42	0.09	—	Encased	Yes	No	Yes	Open lumen	4		
V15	Positive	0.843	0.042	—	50% encased	Mild	No	No	Open lumen	3		

Reference: from Loukeris et al. [39].

*GW* gestational weeks, *V* chorionic villus, *SARS-CoV-2* nucleocapsid, clone BSB-B4, *Circ* circumference, *SCT* syncytiotrophoblast, *SK* syncytial knots, *CHI* chronic histiocytic intervillositis, *NA* non available

### Statistical analysis

For virus replication and host response, a few (2-5, depending on the availability of sufficient cell yield after isolation) technical replicates were tested for each donor. The number of replicates is summarized in Table S2. To avoid overrepresentation of values from the same donors, means of each donor are presented in the corresponding graphs and used for statistical analyses.

For host response induction and FI, each dot corresponds to the mean of values from an individual donor. The average of all donors (biological replicates) and the standard error of mean (SEM) are represented in the corresponding graphs.

Pearson’s correlation test was used to evaluate the associations between host response and viral replication and between FI and virus replication.

In all multiple comparisons, we used Ordinary One-Way ANOVA (Analysis of Variance) followed by Tukey’s Multiple Comparison Analysis. This analysis and the determination of adjusted *p* value (p-val), mean difference and 95% confidence interval (CI) were performed in prism (V10.0.3). Significance level was set at *p* value < 0.05. *p* values: <0.05 (*), < 0.01(**), < 0.001(***) and <0.0001 (****).

## Results

### Permissiveness of STB to SARS-CoV-2 Delta and Omicron variants in vitro

**Fig. 2:**
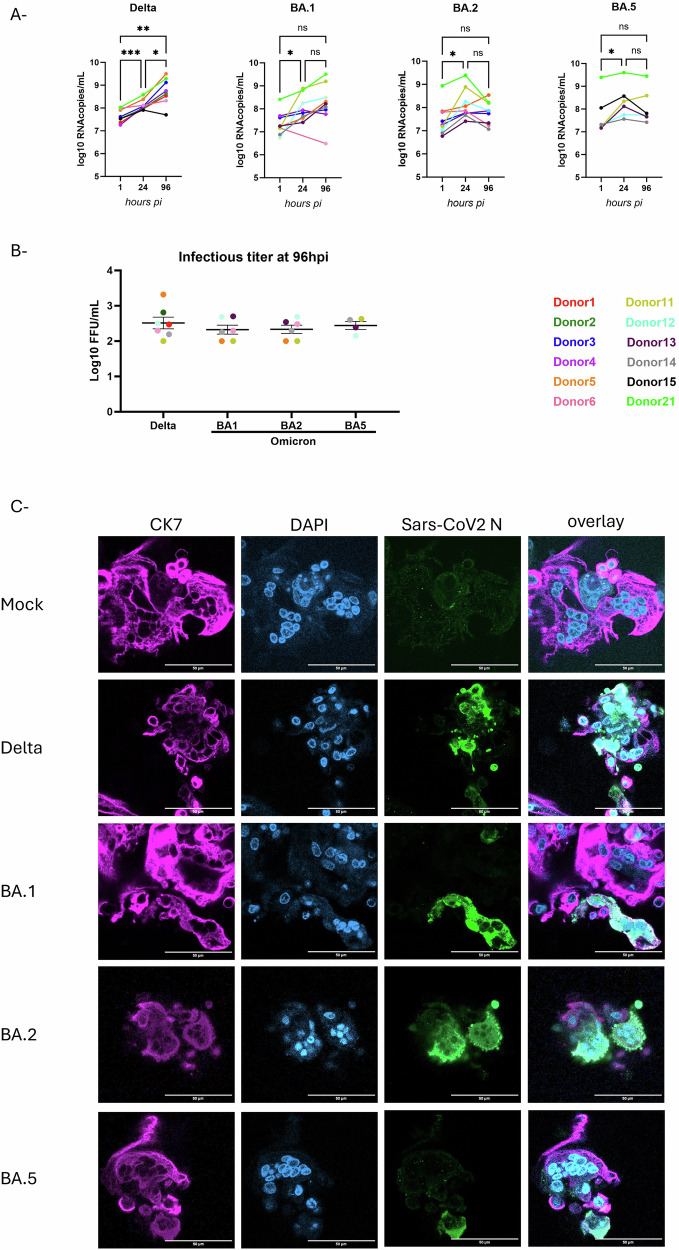
SARS-CoV-2 Delta and Omicron variants are propagating in first trimester STB. CTBs were isolated from first trimester trophoblasts and cultured for 96 h to allow STB formation before being inoculated using clinical isolates of SARS-CoV-2 Delta or BA.1, BA.2 and BA.5 Omicron variants at multiplicity of infection (MOI) of respectively 0.1 and 0.2 for 1 h. **A** SARS-CoV-2 replication. For each time point, the supernatant was collected and replaced by fresh media. Viral RNA was quantified by real time RT-PCR. Statistically significant increase was calculated using 2-way ANOVA. *p* values: <0.05 (*) and <0.001(***). Each symbol represents the average vRNA of values quantified from an individual donor. Hpi: hours post-infection. **B** Viral titers at 4dpi of SARS-CoV-2 variants assessed by focus forming assays. Each symbol represents the average of values (in FFU/mL) quantified from an individual donor. The mean and SEM of all donors are shown in the graph. **C** Immunofluorescence at 4 days post-infection. Mock and SARS-CoV-2 infected cells were fixed, permeabilized, and co-stained using antibodies against SARS-CoV-2 nucleoprotein (SARS-CoV-2 N in green) and cytokeratin 7 (CK7 in pink), a trophoblast cells’ marker. The nuclei were stained by DAPI (blue). Images were acquired using Zeiss LSM 800 Meta confocal microscope with a 63.6/1.4 objective. Scale bar: 50 µm.

To test the susceptibility of first trimester STB to SARS-CoV-2 variants, CTB were cultured 3 days to allow cell fusion and differentiation before being infected with SARS-CoV-2 variants. As shown in Fig. 2A, all donors were permissive to infection by SARS-CoV-2 Delta, with an increase of log10 viral RNA copies/mL (vRNAc/mL) varying from 7.9 to 8.6 at 24 hpi and from 7.7 to 9.1 at 96 hpi. An increase of viral replication of Omicron variants was shown in the majority of, but not all donors, with log10 viral RNA copies/mL (vRNAc/mL) for Omicron BA.1 ranging from 7.4 to 8.9 log10 vRNAc/mL at 24 hpi and 6.5 to 9.5 log10 vRNAc/mL at 96 hpi, for Omicron BA.2 from 7.4 to 9.5 log10 vRNAc/mL at 24 hpi and 7.1 to 8.5 log10 vRNAc/mL at 96hpi, and for Omicron BA.5 from 7.6 to 9.6 log10 vRNAc/mL at 24hpi and 7.4 to 9.5 log10 vRNAc/mL at 96 hpi. Viral RNA increase was mainly observed from 1 hpi (baseline after inoculation and washing) to 24 hpi and from 1 and 96 hpi. These results also showed inter-donor variability regarding SARS-CoV-2 infections. Donors 21, 11, and 12 consistently showed high replication, while donors 14 and 6 consistently showed low replication in terms of production of viral RNA 24 and 96 h after infection.

Viral replication was also confirmed by immunofluorescence at 96hpi that showed the localization of SARS-CoV-2 in STB (Fig. 2B). However, only few STB were infected (not shown). The presence of infectious particles was also assessed by focus forming assays and showed comparable infectious titers at 4dpi, but the highest titers were found in case Delta variant (mean 2.52 Log10 FFU/mL), compared to BA.1(mean 2.39 Log10 FFU/mL), BA.2 (mean 2.43 Log10 FFU/mL) and BA.5 (mean 2.48 Log10 FFU/mL) Omicron variants.

In summary, these results confirm that SARS-CoV-2 is able to infect and replicate in STB with replication efficiency dependent on SARS-CoV-2 variants and placenta donors.

### Assessment of the association between host factors and SARS-CoV-2 infection in STB

**Fig. 3:**
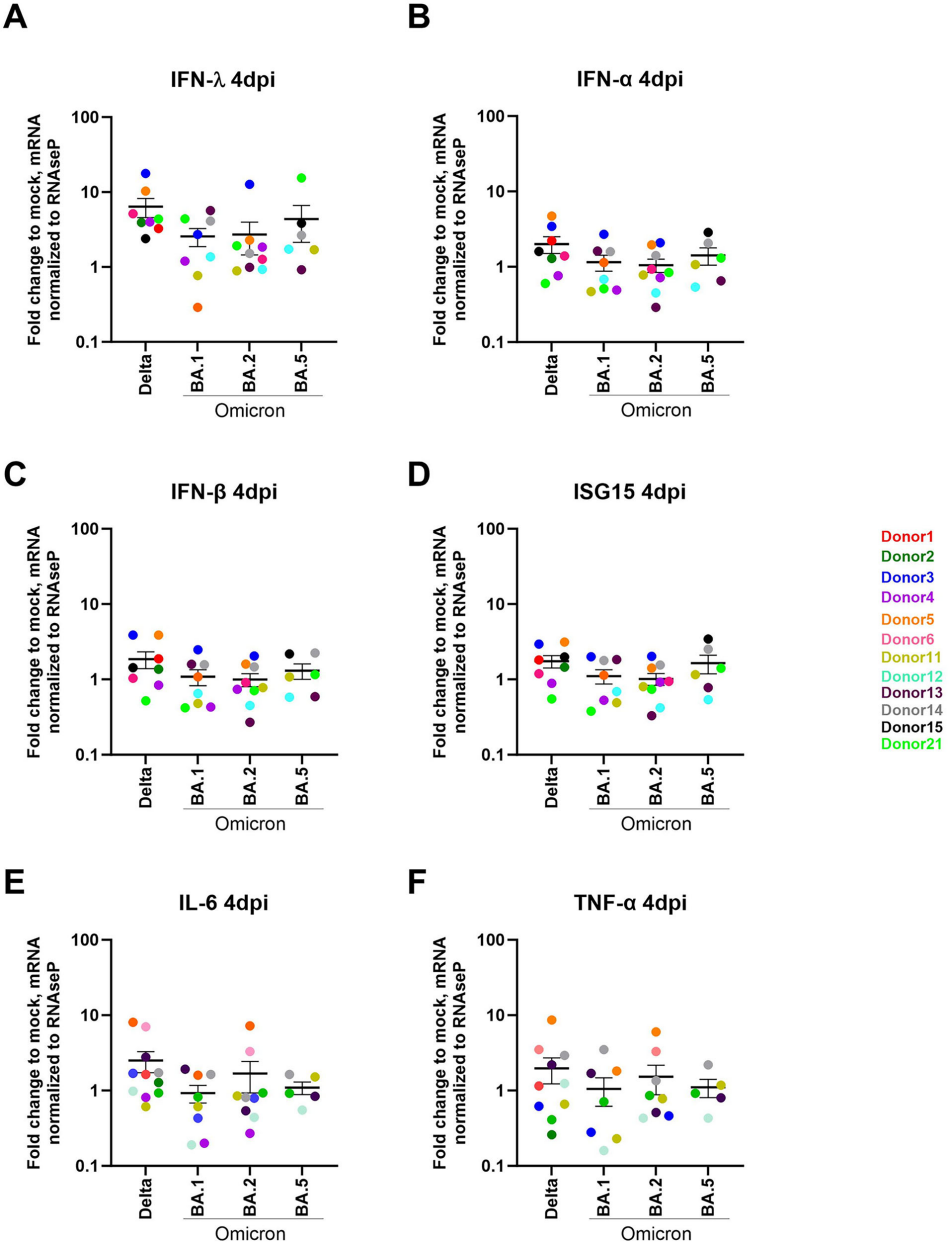
STB responses following SARS-CoV-2 Delta and Omicron variants infection. Induction of IFN-α, IFN-β, IFN-λ, and ISG15 was semi-quantified by real time RT-PCR and represented in fold change relative to non-infected cells and normalized to RNAseP. The mean and SEM of all donors are shown in the graph.

We next assessed innate immune response induction by, first, the determination of fold change expression of genes related to IFN (IFN-α and IFN-β, IFN-λ and ISG15), as the immediate innate immune response induced by respiratory viruses [15, 35, 40] in infected, relative to non-infected STB. Upon STB infections with SARS-CoV-2, we observed a heterogenous host response, including innate immunity and entry host factors, of the trophoblast donors (Fig. 3 and S2). As commonly observed in respiratory virus infections [35, 40, 41], type III interferon, IFN-λ showed a more pronounced response compared to type I, IFN-α and IFN-β (Fig. 3A–C). SARS-CoV-2 Delta infection induced the highest level of interferon response followed by a slight increase of ISG15 (Fig. 3D) expression, while in Omicron infection this response was barely observed. Further statistical analysis showed no correlation (Pearson) between virus replication and the induction of any of these genes related to the innate immune response in STB (data not shown).In addition to IFNs, innate immune response was determined targeting pro-inflammatory cytokines previously assessed in patients and ex-vivo [15]. Weak-to-no inductions (relative to mock-infected STB) of IL-6 and TNF-α were also observed upon SARS-CoV-2 (Fig. 3E, F) by all variants. Nevertheless, a slight induction of IL-6 (mean 4.44-fold increase compared to mock-infected STB) was observed in STB infected with the Delta variant.

**Fig. 4:**
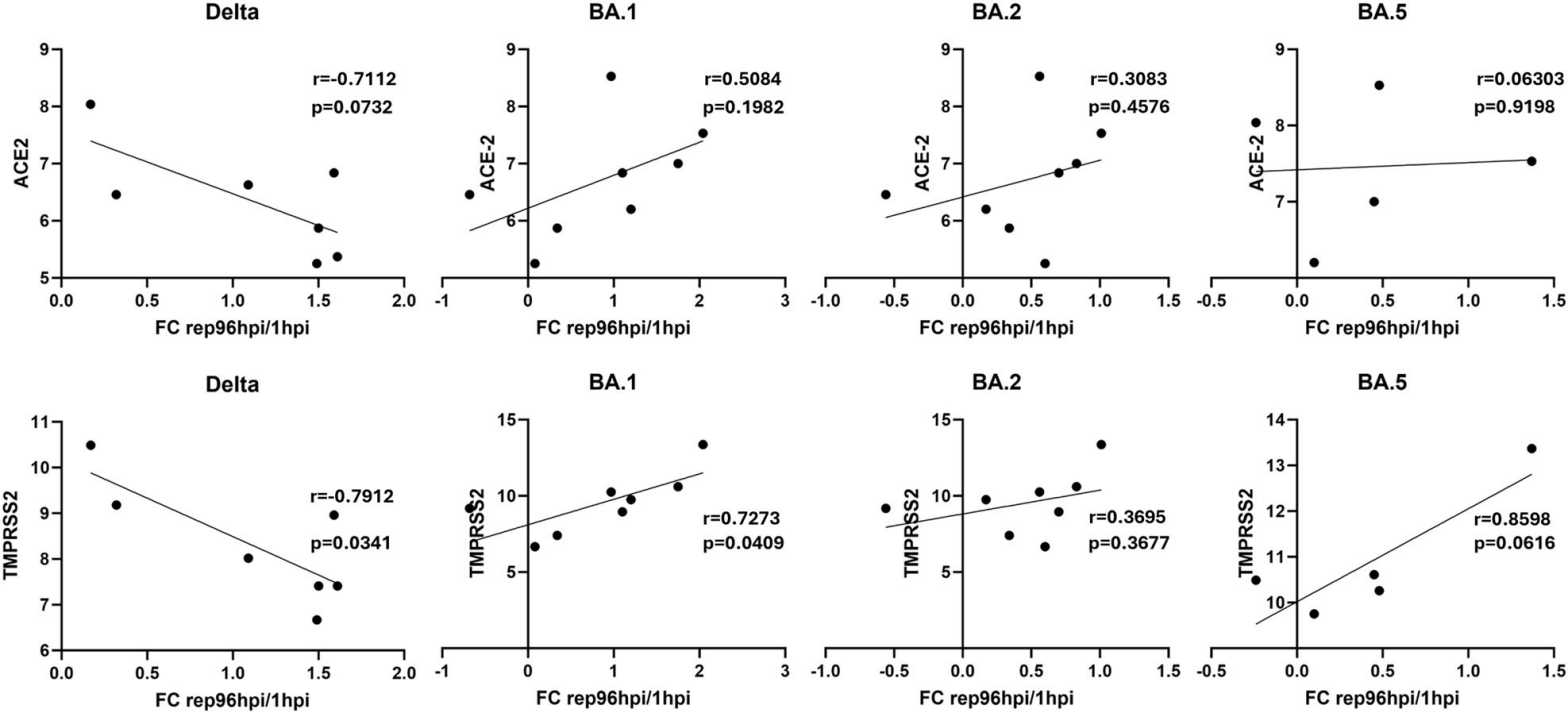
Correlation of SARS-CoV-2 variants replication 96 h post infection of STB and ACE-2 and TMPRSS2 mRNA levels. Associations were tested using Pearson’s correlation test. Each symbol represents the average of values from an individual donor.

We further investigated the induction of host factors involved in virus entry, including SARS-CoV-2 receptor, ACE-2, and the cellular protease involved in fusion, TMPRSS2, by RT-qPCR. Donor-dependent differences in baseline expression of TMPRSS2 were observed (mock infected cells from the corresponding donors) with expression significantly correlating with viral replication of Delta and BA.1 infection (Fig. 4). In contrast, the expression of ACE-2 does not correlate with virus replication. No altered expression of ACE-2 or TMPRSS2 was observed during infection (Figure S2).

### SARS-CoV-2 infection of CTB induces significant increase of syncytia formation compared to control cells in vitro

**Fig. 5:**
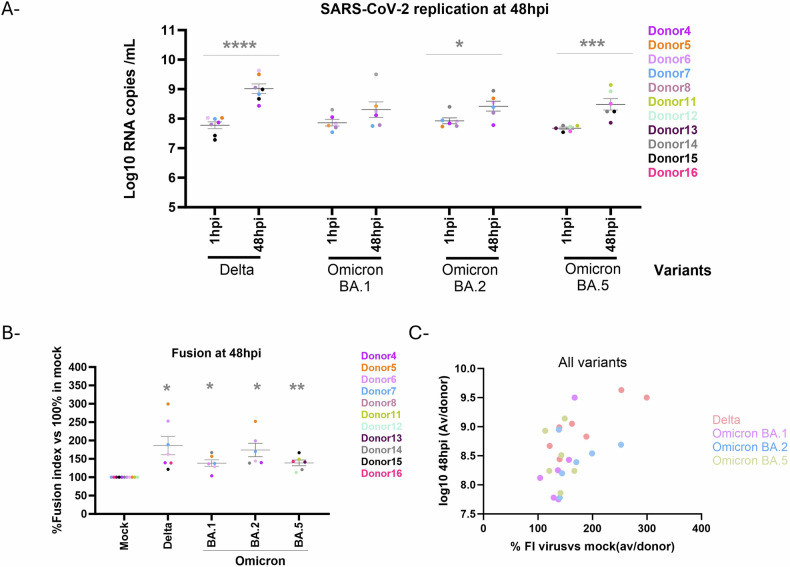
SARS-CoV-2 Delta and Omicron variants replication in first trimester cytotrophoblast cell and effects on cell fusion. **A** SARS-CoV-2 replication. For each time point, the supernatant was collected and replaced by fresh media. Viral RNA was quantified by real time RT-PCR. A statistically significant increase was calculated using 2-way ANOVA **P < 0.01. Each color represents the average values from an individual donor. Each value corresponds to the average of three replicates from the same donor. The mean and SEM of all donors are shown in the graph. **B** Nuclei and syncytia were counted and fusion index was calculated. A statistically significant increase was calculated using 2-way ANOVA. *P < 0.05, **P < 0.01, ***P < 0.001 and ****P < 0.0001. Each color represents the average of three replicates of an individual donor. The mean and SEM of all donors are shown in the graph. **C** SARS-CoV-2 replication was plotted as a function of relative fusion index. Associations were tested using Pearson’s correlation test. Hpi: hours post infection.

**Fig. 6:**
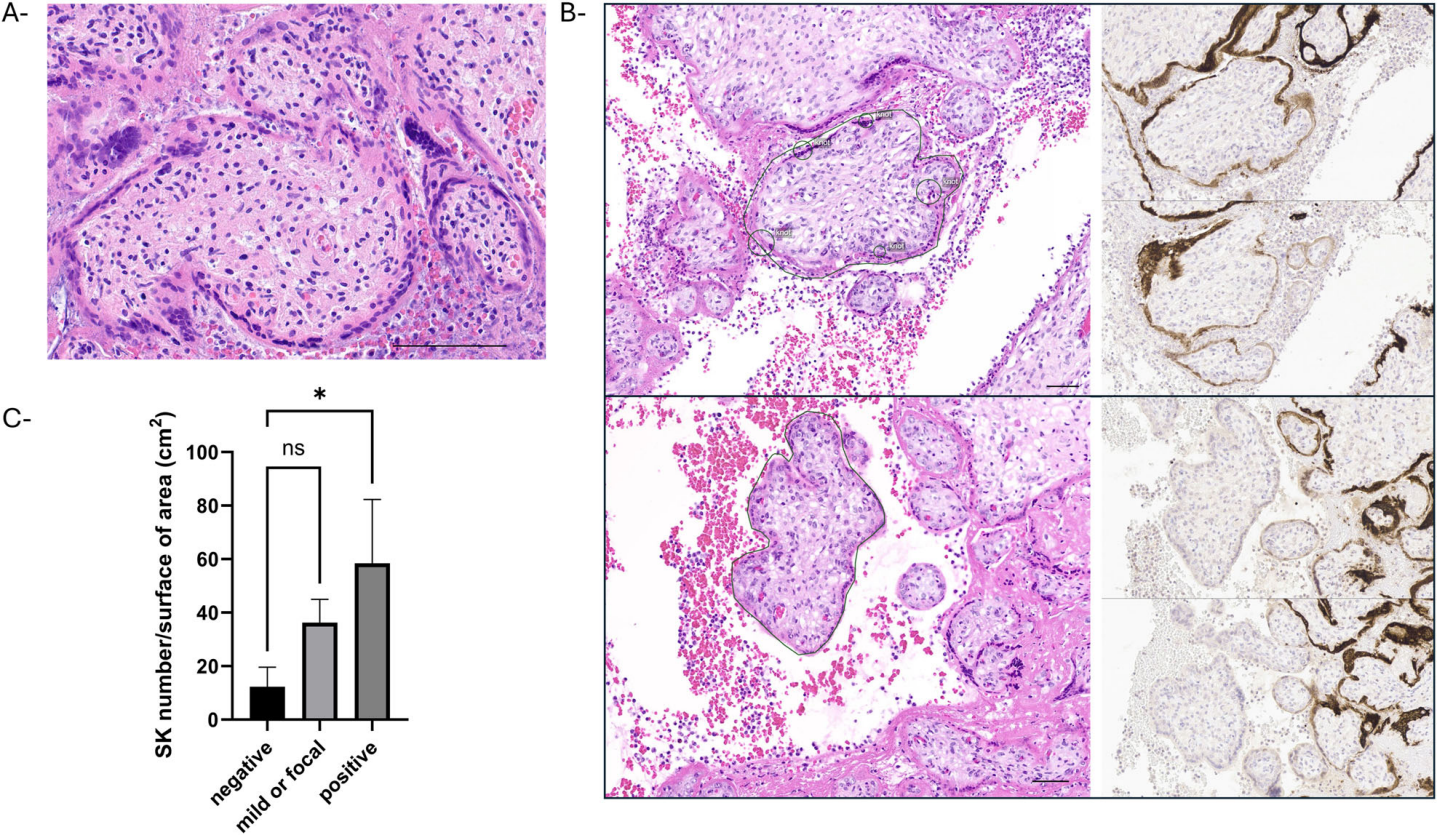
Syncytial knots evaluation depending on reactivity to the SARS-CoV-2 nucleocapsid in 3 early second trimester placenta. Tissue was stained using antibodies against SARS-CoV-2 nucleocapsid (clone BSB-134). SK evaluation was performed on serial H&E placental sections. SK were defined as sessile aggregations of nuclei appearing and disappearing through the serial sections and seen in two or more sections. **A** SK tended to be more numerous in villi encased by fibrin (Case #1, H&E, scale bar = 100 mm). **B** SK release was categorized depending on the SARS-CoV-2 staining on the first and the last serial sections as follows: negative: no reactivity to SARS-CoV-2 NC; mild: focal or weak SARS-CoV-2 staining; positive: SARS-CoV-2 staining is positive throughout the area assessed. Depicted are SK counts in a positive villous showing 5 SK (in circles), and in a negative villous with no SK (Case #3, H&E and SARS-CoV-2 NC, scale bar = 100 mm). **C** The analysis of SK release was then categorized depending on the SARS-CoV-2 staining. Negative: no reactivity to SARS-CoV-2 nucleocapsid; mild: focal or weak SARS-CoV-2 staining; Positive: SARS-CoV-2 staining is positive throughout the area assessed. A statistically significant increase was calculated using one-way ANOVA test. *P < 0.05.

We then investigated the effect of SARS-CoV-2 variants infection on trophoblast cell fusion (Fig. 5). CTB were permissive to SARS-CoV-2 with a significant increase in viral replication between 1 h and 48 hpi for all the variants, except BA.1 (Fig. 5A), reaching an average of 9 log10 vRNAc/mL for Delta variants and averages varying from 8.2 to 8.5 log10 vRNAc/mL for Omicron variants at 2 days pi. The fusion index (FI, Fig. 5B) was also significantly increased in infected cells. The highest FI increase relative to mock-infected CTB was observed in Delta variant-infected cells (adjusted p-val: 0.0287, mean difference: 86.23% increase, 95% CI: 161.7 to 10.80), compared to BA.1 (adjusted *p* val: 0.0149, mean difference: 38.43% increase, 95%CI: 66.66 to 10.21), BA.2 (adjusted *p* val: 0.0184, mean difference: 74.03% increase, 95%CI: 131.2 to 16.83) and BA.5 (adjusted *p* val: 0.0079, mean difference: 38.95% increase, 95%CI: 63.62 to 14.28) Omicron infected cells. As shown in Fig. 6C, this FI enhancement positively correlated with viral replication (Pearson *r* = 0.5466, *p* value = 0.0047). In conclusion, we here demonstrated an efficient infection of SARS-CoV-2 in CTB leading to an increase in cell fusion, which corroborates the noted inter-donor variability.

### SARS-CoV-2 placenta in vivo is associated with an early release of syncytial knots

An increased SK release was previously reported in third trimester placenta of COVID-19 infected patients suggesting that SARS-CoV-2 infection could alter STB turnover [26, 42]. To check whether SARS-CoV-2 infection could alter STB turnover during earlier stage of pregnancy in vivo, we retrospectively evaluated SK release of three early second trimester placenta from COVID19+ pregnant patients (see Table 1). Detection of SK was compared in 3 different areas of the sections, depending on reactivity to the anti-SARS-CoV-2-N (Fig. 6B). As shown in Fig. 6C, a significant increase in SK in SARS-CoV-2-positive areas of placenta was observed compared to SARS-CoV-2- negative areas from the same placenta (adjusted p-val: 0.0231, mean difference: 46.23 SK/cm^2^ of area, 95%CI: 84.40 to 8.05), suggesting an association between SARS-CoV-2 infection and SK early release. The percentage of villi showing one or more SK was also much higher than expected during the second trimester (35% vs. reference numbers of 5.5% at 21 GW; 43% vs. 8.6% at 24 GW; 34%, no reference data available at 18 GW). SK tended to be seen more frequently on villi encased by fibrin (Fig. 6A).

## Discussion

Placental formation, development, and function are crucial for both maternal and fetal health during pregnancy. SARS-CoV-2 infections during pregnancy have been associated with placental injury and clinical adverse outcomes [12, 43, 44]. Recent trending SARS-CoV-2 studies were mainly focused on vaccination, management, pregnancy outcomes, and transmission to the fetus [7]. Studies on the fetus’ susceptibility to SARS-CoV-2 predominantly targeted later stages of pregnancy [5, 8, 9]. While of strong interest, questions remain regarding the susceptibility of fetuses to SARS-CoV-2 at the earliest stages of pregnancy. This is mainly due to the difficulty in obtaining clinical samples, from infected SARS-CoV-2 donors and who voluntarily interrupt their pregnancies at this stage, to participate in in vivo or in vitro studies respectively. Alternatively, stem cell-based system was used to evaluate the effects of SARS-CoV-2 infection in early gestation [16, 30, 45]. STB derived from human embryonic stem cells (hESC), human expanded potential stem cells or induced (i) or placenta-derived trophoblast stem cells (TSC) express the entry host factors ACE-2 and TMPRSS2 and support replicative infection by SARS-CoV-2 [16, 30, 45]. TSC-derived STB infected with SARS-CoV-2 also elicits an interferon-mediated immune response [45] suggesting that placental development could be altered by early SARS-CoV-2 infection.

By using a relevant 2D first trimester trophoblast model and clinical viral isolates, we demonstrated that first trimester CTB and STB are permissive to SARS-CoV-2 variants. Supporting assumptions that Delta variant contributes to adverse pregnancy outcomes [8], our study findings confirmed the high increase in viral replication of the Delta variant shown in most donors. Omicron variants BA.1, BA.2, and BA.5 also showed an increase in viral replication, yet not as substantial as the Delta variant. In line with viral replication, host response, including the induction of IFNs and the pro-inflammatory cytokine IL-6, was slightly higher in STB infected by the Delta variant. This is consistent with the clinical observation reporting higher rate of hospitalization in Delta- compared to Omicron-dominant waves of SARS-CoV-2 [46], which could indicate intrinsic higher pathogenicity of Delta. It remains to be considered though, that also population immunity was lower in times of Delta virus circulation versus Omicron. Since the emergence of Omicron in late 2021, the COVID-19 pandemic has entered a new phase, marked by the continued evolution of SARS-CoV-2 into multiple lineages within the Omicron clade. However, no increased clinical outcomes have been recorded with the more recent Omicron variants in patients.

Our data is coherent with the outcomes from a study conducted in term placental section explants reporting similar conclusions in terms of virus replication efficiency (infectious titer assessment), inter-donor variability and induction of genes related to IFN and inflammation, namely IL-6 and TNF-α [15]. The lower efficiency of replication in placenta cells compared to human airway epithelium [36] may result from a combination of factors, including a restricted expression of viral entry receptors such as ACE-2 and TMPRSS2, as well as the unique innate immune features of the placenta. This immune environment is adapted to maintain maternal-fetal tolerance and may modulate, rather than amplify, classical antiviral responses to avoid excessive inflammation that could compromise fetal development.

In contrast to the observation made on term placenta [15], trophoblast expression of ACE-2 does not correlate with SARS-CoV-2 replication in first trimester STB derived from iTSCs [16]. However, TMPRSS2 expression significantly correlates with Delta and BA.1 replication. We observed the same tendency with BA.5 replication, but not with BA.2 replication. This observation suggests that TMPRSS2 may play an important role in some SARS-CoV-2 variants entry to the STB. Since TMPRSS2 was previously shown to accelerate SARS-CoV-2-mediated cell-cell fusion [47], we next investigated the effect of SARS-CoV-2 infection on trophoblast cell fusion. An increase in trophoblast cell fusion was observed in trophoblast cells infected with SARS-CoV-2 and correlates with viral replication. The increase in cell fusion is higher in cells infected with Delta variant compared to cells infected with BA.1 variant, suggesting a lower fusogenicity of Omicron BA.1 than Delta, as already described [48–50]. These results also suggest that the SARS-CoV-2 infection could accelerate trophoblast cell fusion and STB turnover. This hypothesis corroborated clinical analysis of early placenta infected by SARS-CoV-2, showing higher detection of SK release in SARS-CoV-2-positive, compared to negative, areas from the same placenta. Indeed, SK are generally considered as structures involved in the elimination of aged or damaged areas of the STB, and their accumulation is classically interpreted as a hallmark of increased trophoblast turnover. In our study, the higher number of SK in SARS-CoV-2-infected villi compared to adjacent non-infected villi from the same placenta provides strong evidence for a localized increase in STB turnover, limiting potential inter-individual confounders. Together with the enhanced trophoblast FI observed in vitro upon infection, these findings suggest an accelerated renewal of STB to compensate the shedding process of the STB in response to SARS-CoV-2 infection and thus maintain STB homeostasis. In addition, a number of studies had previously reported an association between SK increase and adverse obstetrical outcomes such as fetal growth restriction, preeclampsia, and stillbirth [51]. It is therefore tempting to speculate that this local placental response to SARS-CoV-2 infection may contribute to the obstetric complications reported mainly during the Delta variant wave. The higher trophoblast cell fusion observed in vitro is thus coherent, not only with the pathological analysis of SARS-CoV-2 infected placenta, but also with previous patients’ reports [26–28]. However, further studies are required to demonstrate a causal link between SARS-CoV-2-induced placental alterations and clinical outcomes.

Nevertheless, these observations are not in accordance with previous studies using human trophoblast stem cells (hTSC) and determining the inhibitory effect of SARS-CoV-2 infection on STB differentiation [16, 30]. These discrepancies could be due to the different trophoblast models used for in vitro infection and syncytialisation studies [31]. Primary first trimester trophoblasts are widely recognized as a robust model for investigating trophoblast fusion. Trophoblast stem cell-derived placental model is interesting in studying infection during earlier stages. Mezzano et al. identified early STB (eSTB) with the TSC-based system model, which is undetectable in first trimester primary trophoblast but more susceptible to SARS-CoV-2 infection than STB [30]. In this model, eSTB infected with SARS-CoV-2 may decrease STB maturation. In any cases, it has been shown that SARS-CoV-2 infections could alter the STB renewal and/or turnover and consequently the integrity and function of STB, which in turn may impact the fetal-maternal exchanges as well as hormones secretion during pregnancy.

Altogether, infecting 2D first trimester trophoblast and analyzing early second-trimester infected placenta from fetal demise, filled gaps in the understanding of SARS-CoV-2 placenta infections and potential mechanisms involved in complications related to COVID-19 during pregnancy. The strength of our findings is the concordance between in vitro and clinical findings, which highlights the relevance of our model system when studying the effect of viral infection on trophoblastic cell fusion and virus host interactions. Such a model does not only serve to better understand SARS-CoV-2 impact in pregnancy but could also be used as an in vitro risk assessment tool for other emerging viruses and their impact during early pregnancy.

## Supplementary information

Supplementary tables

Supplementary Figure 1

Supplementary Figure 2

## Data Availability

The data that support the findings of this study are available on request from the corresponding author.
