# Supplementary material for: SARS-CoV-2 delta and omicron variants alter trophoblast cell fusion and syncytiotrophoblast dynamics: new insights into placental vulnerability
Source: Cell Death Dis. 2025 Oct 7;16(1):718. doi: 10.1038/s41419-025-08016-x (PMC12504560; doi:10.1038/s41419-025-08016-x)
Supplement: Supplementary file 1 — Supplementary tables [file 41419_2025_8016_MOESM1_ESM.docx]

**Table S1.** Information about virus isolates. MOI: Multiplicity of infection. vRNAc: viral RNA copies quantified from the inoculum corresponding to the respective MOI in 24-well plate.

|  | **Virus name** | **GISAID accession ID** | **Clade** | **reference** | **Pango lineage** | MOI | vRNAc/100ul of inoculum |
| --- | --- | --- | --- | --- | --- | --- | --- |
| **Variant** |  |  |  |  |  |  |  |
| Delta | hCoV19/Switzerland/GE33896105/2021 | EPI_ISL_1811202 | AY.122 | Bekliz et al., Nat Com 2022 | GK | 0.1 | 1.06E+09 |
| Omicron BA.1 | hCoV-19/Switzerland/VD-HUG-36221084/2021 | EPI_ISL_7605546 | GRA | Bekliz et al, BioXriv 2024 | BA.1 | 0.2 | 1.81E+10 |
| Omicron BA.2 | hCoV-19/Switzerland/un-HUG-36637889/2022 | EPI_ISL_8680372 | GRA | Bekliz et al, BioXriv 2024 | BA.2 | 0.2 | 8.75E+09 |
| Omicron BA.5 | hCoV-19/Switzerland/GE-HUG-37911771/2022 | EPI_ISL_12695073 | GRA | Bekliz et al, BioXriv 2024 | BA.5.1 | 0.2 | 4.57E+10 |

**Table S2:** Summary of Placenta cells used from anonymized donors. After isolation and purification and cultured into cytotrophoblast (CTB) or syncytiotrophoblast (STB) cells. The gestational week (GW) is represented in column 2 (day, d). Pool: pool of donors. Each donor was tested in n technical replicates for each virus (indicated between” ()” in the right column). The number of technical replicates was variable depending on the yield of isolated placenta cells. Different experiments were performed for each donor: infections were analysed to assess virus replication (VR), host response induction (HRI), fusion index (FI) or by immunofluorescence (IF).

|  | **GW** | **STB/CTB** | **Analyses** | **virus tested (number of replicates/virus)** |
| --- | --- | --- | --- | --- |
| **Donor1** | 11GW + 2d | STB | VR + HRI +IF | Delta (5) |
| **Donor2** | 11GW + 3d | STB | VR + HRI | Delta (4) |
| **Donor 3** | pool 10GW + 10GW+6d + 10GW+3d | STB | VR + HRI | Delta (3), BA1 (3) and BA2 (3) |
| **Donor 4** | 10GW + 2d | CTB | FI | Delta (3), BA1 (3) and BA2 (3) |
|  |  | STB | VR + HRI | Delta (3), BA1 (3) and BA2 (3) |
| **Donor5** | 11GW + 1d | CTB | FI +VR | Delta (3), BA1 (3) and BA2 (3) |
|  |  | STB | VR + HRI | BA1 (2) and BA2 (2) |
| **Donor6** | 11GW + 2d | CTB | FI +VR | Delta (3), BA1 (3) and BA2 (3) |
|  |  | STB | VR + HRI | BA1 (2) and BA2 (2) |
| **Donor7** | 11GW + 4d | CTB | FI +VR | Delta (3), BA1 (3) and BA2 (3) |
| **Donor8** | 11GW + 6d | CTB | FI +VR | Delta (3), BA1 (3) and BA2 (3) |
| **Donor 11** | 9GW | CTB | FI +VR | Delta (3) and BA5 (3) |
|  |  | STB | VR + HRI +IF | BA1 (2), BA2 (2) and BA5 (2) |
| **Donor 12** | 10GW + 2d | CTB | FI +VR | BA5 (3) |
|  |  | STB | VR + HRI | BA1 (2), BA2 (2) and BA5 (2) |
| **Donor 13** | 10GW + 4d | CTB | FI +VR | BA5 (3) |
|  |  | STB | VR + HRI | BA1 (2), BA2 (2) and BA5 (2) |
| **Donor 14** | 10GW + 6d | CTB | FI +VR | BA1 (3), BA2(3) and BA5 (3) |
|  |  | STB | VR + HRI | BA1 (4), BA2 (4) and BA5 (4) |
| **Donor 15** | 10GW + 5d | CTB | FI +VR | BA5 (3) |
|  |  | STB | VR + HRI | Delta (3) and BA5 (3) |
| **Donor 16** | 10GW | CTB | FI +VR | Delta (3) and BA5 (3) |
| **Donor21** | pool 10GW + 11GW | STB | VR + HRI + IF | Delta (2), BA1 (2), BA2(2) and BA5 (2) |
