# Supplementary figures and images for: SARS-CoV-2 delta and omicron variants alter trophoblast cell fusion and syncytiotrophoblast dynamics: new insights into placental vulnerability
Source: Cell Death Dis. 2025 Oct 7;16(1):718. doi: 10.1038/s41419-025-08016-x (PMC12504560; doi:10.1038/s41419-025-08016-x)

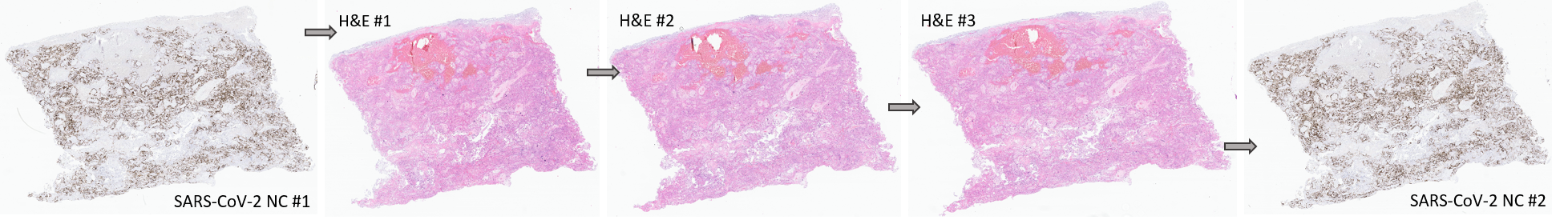

Supplement: Supplementary file 2 — Supplementary Figure 1 [file 41419_2025_8016_MOESM2_ESM.tif]

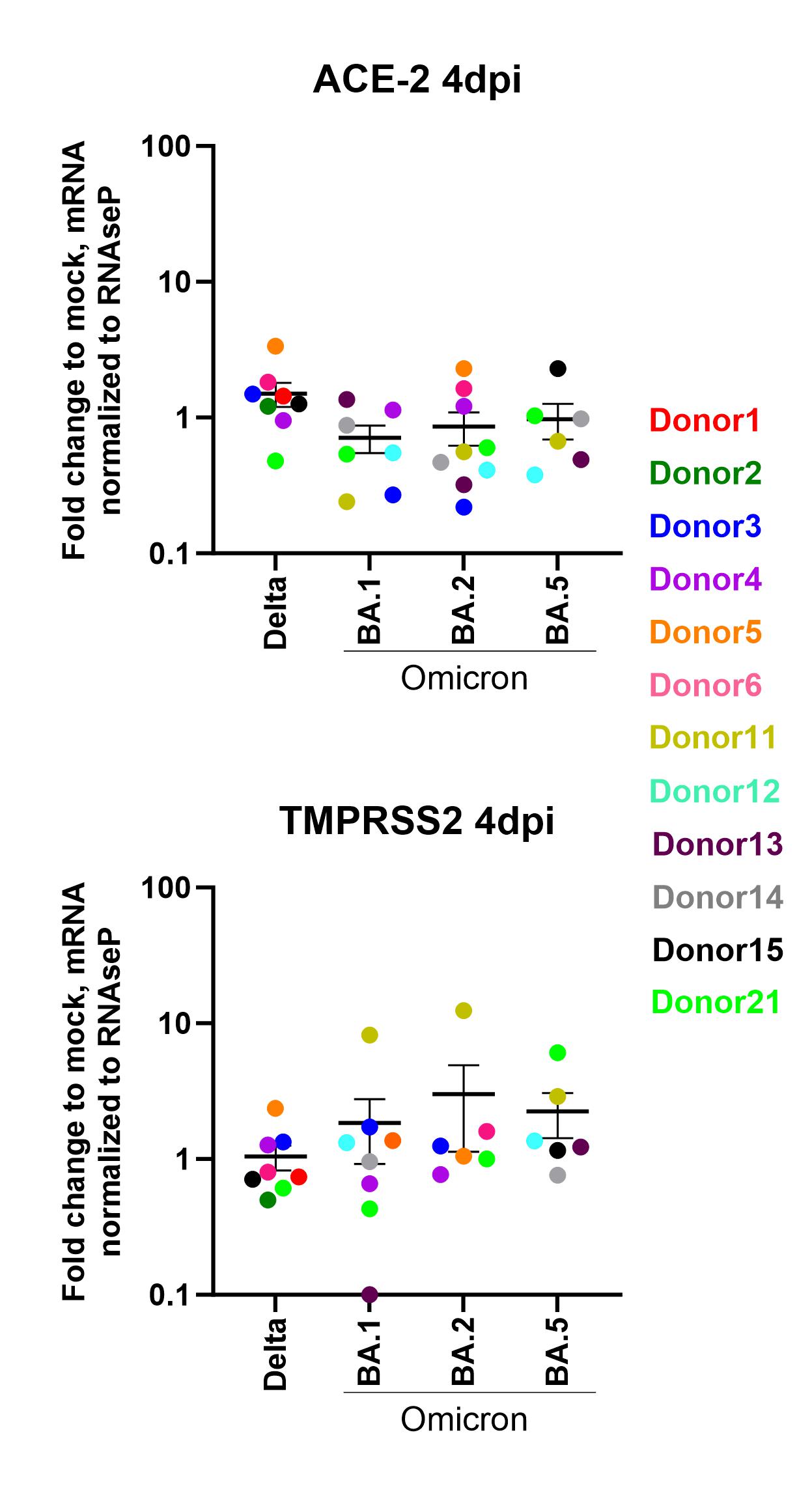

Supplement: Supplementary file 3 — Supplementary Figure 2 [file 41419_2025_8016_MOESM3_ESM.jpg]
